# Cerebral venous sinus stenting and jugular bulb embolization for pulsatile tinnitus: A case report

**DOI:** 10.3389/fneur.2024.1330619

**Published:** 2024-02-05

**Authors:** Mengjiao Xu, Xiaobo Dong, Can Zheng, Tao Zheng, Gesheng Wang

**Affiliations:** Department of Brain Disease III, Dongfang Hospital, Beijing University of Chinese Medicine, Beijing, China

**Keywords:** pulsatile tinnitus, jugular bulb, sigmoid sinus, stenting, spring coil embolization

## Abstract

**Background:**

Pulsatile tinnitus (PT) is a rare form of tinnitus that aligns with the heartbeat. It is typically brought on by lesions with significant vascularity, which produce aberrant sound conduction and increase the risk of mental health issues and hearing loss. Venous PT is more prevalent than arterial PT. Open procedures or interventional procedures can be used to treat PT. We present here a case of PT caused by venous luminal stenosis combined with jugular bulb (JB) malformation, which was improved by stenting and JB embolization.

**Case presentation:**

A 59-year-old woman presented with long-term tinnitus consistent with heart rhythm and hearing loss, accompanied by anxiety, insomnia, and depression. The results of brain MRV, CT, and DSA showed stenosis of the right sigmoid sinus and high jugular bulb (JB) with dehiscence of the JB wall. The patient saw a significant improvement in PT symptoms following sigmoid sinus stenting and spring coil embolization of the high JB, following the diagnosis of PT. The patient had no PT recurrence for the course of the 31-month follow-up period.

**Conclusion:**

In the present PT case, there was a simultaneous onset of the right sigmoid sinus stenosis and the high JB with the JB wall abnormalities. Sigmoid sinus stenting and spring coil embolization of high JB may be a treatment for the PT, but the prevention of post-stenting complications is still an issue that requires great attention and needs further study.

## 1 Introduction

Pulsatile tinnitus (PT) is a form of tinnitus characterized by the perception of a rhythmic sound that can be attributed to various identifiable causes (1). This condition typically manifests unilaterally and is caused by alterations in vascular hemodynamics or abnormal conduction of normal sounds. PT can have a significant impact on the patient's overall well-being, often leading to sleep disturbances, anxiety, depression, and in extreme cases, suicidal ideation (2). Furthermore, PT can serve as an early indicator of an underlying and potentially serious medical condition.

Common vascular causes of PT include arterial anomalies, venous anomalies, and arteriovenous fistulae, among which venous PT(VPT) includes idiopathic intracranial hypertension(IIH), venous sinus stenosis(VSS), sigmoid sinus wall abnormalities(SSWA), jugular Vein and other venous malformations (3). VPT can be ameliorated by open procedures such as surface reconstruction/plastic surgery or endovascular interventions such as stenting (4). However, the relatively few reports and pathologic knowledge of venous PT predispose to misdiagnosis and underdiagnosis of PT, which in turn affects therapeutic decisions. Therefore, the etiology and imaging diagnosis of venous PT is crucial (5).

In this case study, we encountered a patient who experienced PT as a result of both VSS and high jugular bulb(JB) issues. The patient underwent successful treatment through the implantation of a sigmoid sinus stenting and subsequent JB embolization. Notably, there was no recurrence of symptoms during the 31-month follow-up period. This report presents the first documented instance of the successful combination of sigmoid sinus stent implantation and JB embolization for the treatment of PT. We report this case in the hope of giving neurosurgeons some references about the diagnosis and treatment of VPT.

## 2 Case description

The patient, a 59-year-old female, presented with bilateral symmetrical low-key tinnitus and accompanying hearing loss three years ago. Over the past year, she experienced a progressively worsening blowing or running sound in her right ear, resembling a “murmur,” along with synchronous with her heartbeat rhythm. Concurrently, her original symptoms worsened, manifesting as slightly decreased visual acuity, insomnia, anxiety, depression, and suicidal ideation. She had previously been diagnosed with sudden deafness and received microcirculation treatment but did not experience any improvement.

The patient reported that the persistent tinnitus in his right ear was seriously affecting his normal life and urgently needed treatment. The patient had suffered from hypertension and hyperlipidemia in the past and was taking medication regularly to keep his blood pressure and blood lipids within normal limits, with no history of other illnesses, no history of allergies to medications or food, and no history of such hereditary diseases in his family. We assessed the patient's tinnitus, sleep and anxiety levels using the Tinnitus Handicap Inventory(THI), the Pittsburgh Sleep Quality Index (PSQI) and the Hamilton Depression Scale(HAMD), with the following results: THI: 56/100 points (Grade 3); PSQI: 14/21 points; and HAMD: 10 points, confirming moderate tinnitus handicap, sleep disorder, and mild anxiety state.

**Figure 1:**
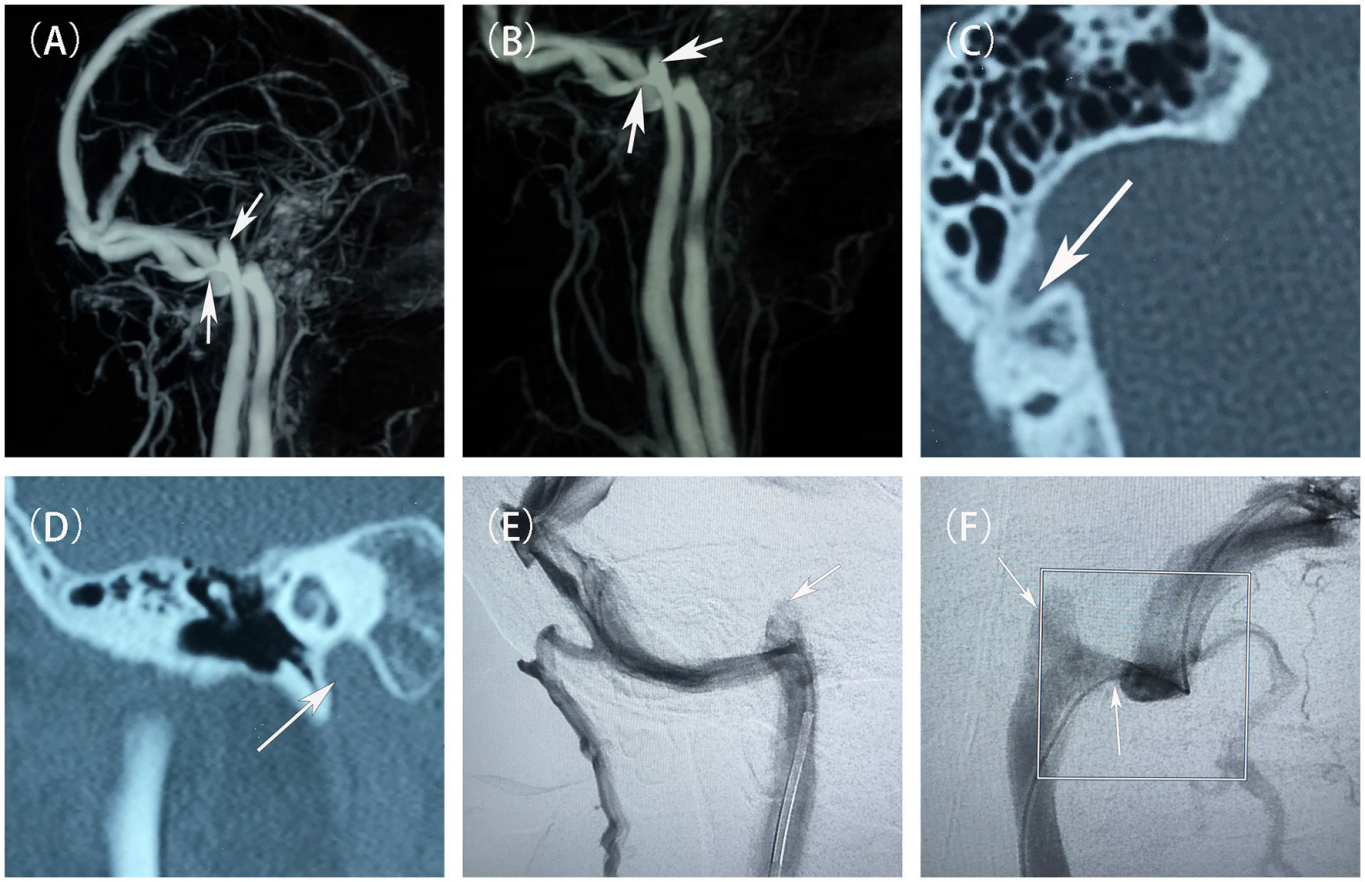
Preoperative evaluation and diagnostic imaging of the patient. **(A, B)** Pre-treatment MRV scans showed the stenosis at the junction of the right sigmoid sinus and the internal jugular vein, and a high jugular bulb (white arrow). **(C)** Pre-treatment non-contrast CT scans showed a branch of the mastoid vein emanating from the right sigmoid sinus. **(D)** Pre-treatment non-contrast CT scans demonstrated the right high jugular vein bulb invaded the inner ear structures. **(E, F)** Pre-treatment DSAs showed that a narrowing of the sigmoid sinus at its junction with the internal jugular vein, and a high jugular bulb (white arrow).

After admission, we performed neurological examinations: (1) intracranial pressure was normal; (2) otoscopy: bilateral external auditory canals were patent, tympanic membranes were intact, grayish-white in color, and no congestion or fluid flatness was seen; the hearing loss was observed in both ears by pure tone audiometry, and tympanic ventricular conductance mapping showed a pattern of 3C; (3) A computed tomography (CT) scan of the right ear showed: normal right sigmoid sinus wall and stenosis at the junction of the right internal jugular vein(IJV) and the sigmoid sinus, as well as a high JB on the right accompanied with Jugular bulb wall dehiscence (JBWD) (Figure 1). Digital subtraction angiography (DSA) showed moderate-to-severe sigmoid sinus stenosis(SSS) at its junction with the IJV (stenosis of approximately 50%–70%) and a high JB (Figure 1), and distal venous sinus manometry was performed. Initially, we thought that the PT in the right ear might be due to the SSS increasing the blood flow velocity creating a vortex in the high JB, so that the PT sound entered the inner ear. Two other evidences supported our diagnosis: first, during DSA venography, the contrast catheter passed through the stenosis site, and when the pressure was measured at the distal end, it changed the direction of blood flow at the site, and the venous vortex flow was reduced, and the patient's PT disappeared; second, by the right side neck-pressure test, when the pressure was increased to a certain degree, the PT disappeared on the patient's right side after the venous reflux was blocked, and the left side hearing was not changed. However, the results of subsequent interventions confirmed more than just what was previously stated.

## 3 Therapeutic intervention

**Figure 2:**
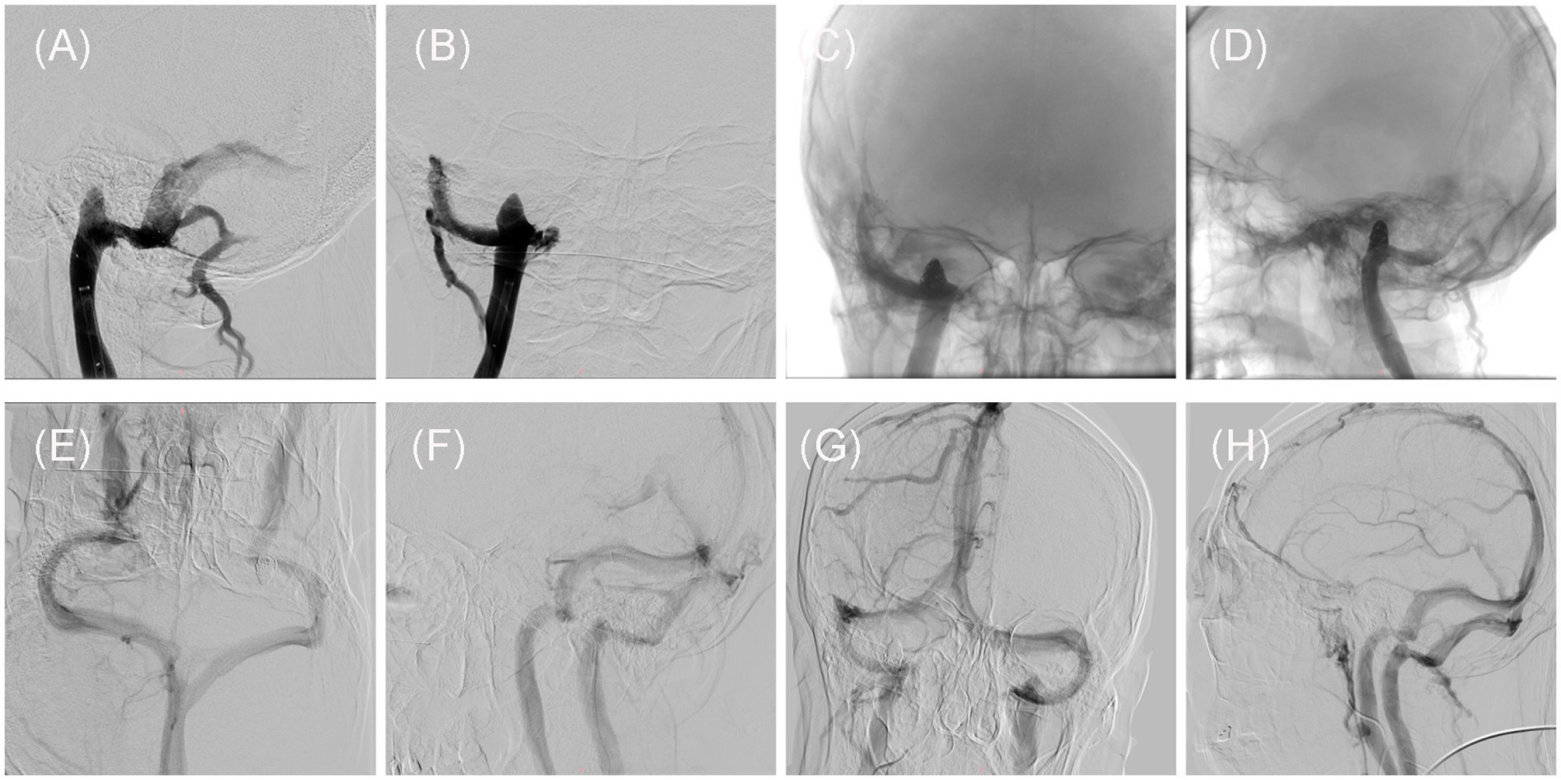
Intraoperative and post-treatment follow-up DSAs of the patient. **(A, B)** Intraoperative DSA showed that the stenosis has been improved by placement of the SAB stent, but the high jugular bulb remains on Dec. 28, 2020. **(C, D)** Intraoperative DSA demonstrated the presence of the spring coils within the high jugular bulb, on Jan. 04, 2021. **(E, F)** The first follow-up DSA showed that the stent at stenosis and the high jugular bulb embolized by the spring coils, on Jan. 17, 2022. **(G, H)** The second follow-up DSA showed that the stent at stenosis and the high jugular bulb embolized by the spring coils, on Aug. 14, 2023.

Following discussions among the neurosurgeons, it was determined that the initial course of action should involve endovascular treatment of the SSS. DSA was performed under local anesthesia to confirm the presence of severe stenosis at the junction of the right sigmoid sinus and the IJV, including a right high JB as depicted in Figure 2. A 6F Long Sheath (Penumbra, California, USA) was inserted into the right femoral vein and positioned at the beginning of the right IJV, below the high JB. The stenosis was successfully crossed using a Synchro 0.014-in micro guidewire (Stryker, Michigan, USA), followed by the placement of a Neuro RX 2.50 × 12 mm balloon (Sinomed, Tianjin, China) across the stenosis. The balloon was then inflated to a pressure of 6 atm, resulting in an improvement of the stenosis. Subsequently, the balloon was withdrawn and a Solitaire AB 6 × 30 mm stent (Medtronic, Minnesota, USA) was deployed at the target site through an Excelsior XT-27 microcatheter (Stryker, Michigan, USA). Post-stenting DSA revealed the disappearance of the stenosis, with contrast-medium observed in the right high JB as shown in Figure 2. The patient reported a reduction in post-treatment pain intensity by approximately 70% to 80%.

Regrettably, the patient experienced a recurrence of PT on the third day following the stenting procedure. The second DSA showed no thrombosis in the stent at the sigmoid sinus, good improvement of stenosis, and no recurrence of stenosis with smooth blood flow (Figure 2). Therefore, we considered that even if the SSS was improved, the blood flow velocity in the sigmoid sinus was only transiently improved, eddy currents still appeared in the high JB, and the jugular bulb wall rupture due to the high JB caused the patient's PT to recur. To address this, a second intervention known as stent-assisted spring coiling embolization was performed. The procedure involved the use of general anesthesia and a 6F Long Sheath (Penumbra, California, USA) to introduce an Excelsior SL-10 microcatheter (Stryker, Michigan, USA) into the right transverse sinus, confirming that the microcatheter was located in the sigmoid sinus stent. A Precise RX 7 × 40 mm stent (Cordis, Florida, USA) was then deployed at the junction of the right sigmoid sinus and the IJV along a Transend 300 cm guidewire (Stryker, Michigan, USA). The microcatheter was adjusted to enter the high JB, where an 8 × 30 mm spring coil (Stryker, Michigan, USA) was positioned to create a basket-like structure. Subsequently, four intracranially detachable spring coils (8 × 40 mm, 7 × 40 mm, 6 × 60 mm, 6 × 20 mm, Gachy, Shanghai, China) were introduced to embolize the High JB. Post-intervention DSA imaging revealed smooth blood flow within the stent and no contrast medium in the right high JB (Figure 2). The patient reported the disappearance of their PT after awakening from anesthesia. Postoperative anticoagulation treatment was administered, consisting of Dabigatran (Boehringer-Ingelheim, Shanghai, China) 150 mg twice daily for the first 6 months, followed by a reduced dosage of 75 mg for the subsequent 6 months.

## 4 Follow-up and outcomes

**Figure 3:**
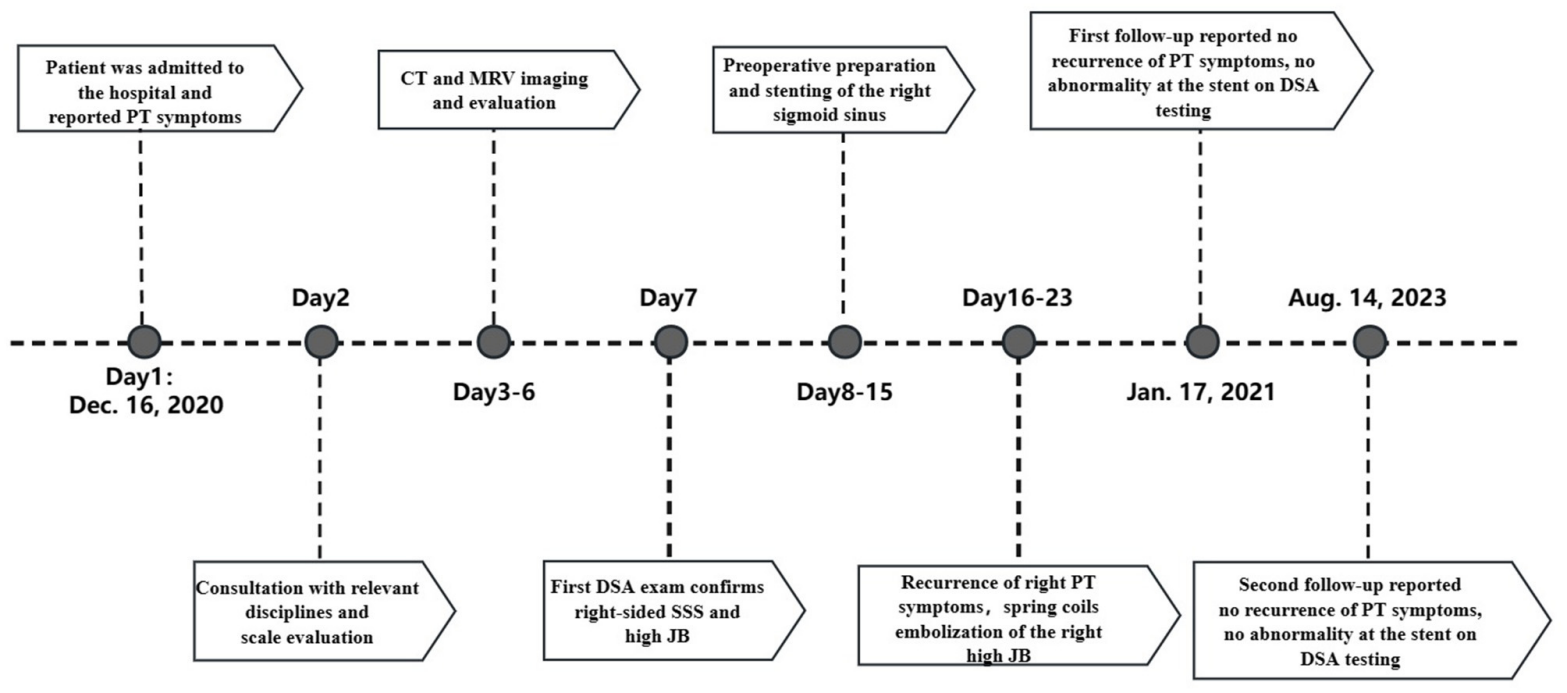
The timeline of the present case.

At the 12-month and 31-month follow-up examinations, the patient‘s right PT has not been recurrent. Both DSA images demonstrated unobstructed blood flow within the stent and complete occlusion of the high JB (Figure 2). THI scores at the two follow-ups were 12/100 (Grade 1) and 10/100 (Grade 1). The timeline of the present case (Figure 3).

## 5 Discussion

Causes of synchronous PT can be categorized as vascular or non-vascular. Vascular causes can be further divided into arterial, venous, and arteriovenous causes. Examples of vascular causes include carotid artery aneurysms, dural venous sinus anomalies, and arteriovenous fistulas. Venous abnormalities are more commonly associated with PT than other etiologies (3). Approximately 10% of PTs are attributed to venous lumen (or sinus) abnormalities (5). In a study by Walters, venous abnormalities were found in 11 out of 18 PT patients, including high or dehiscent jugular bulbs, dural venous sinus anomalies, and venous thrombosis. Compression of the ipsilateral IJV can improve PT symptoms and indicate an association with venous anomalies (6).

In this particular case, the patient initially reported PT with a heartbeat rhythm, suggesting a possible association with vascular abnormalities. After conducting a right neck compression test and DSA, we believe that the patient's PT may be due to SSS. Unilateral SSS usually causes ipsilateral PT, and it would be interesting to find a papillary bone defect on CT (3), whereas SSWA is often associated with stenosis of the sigmoid sinus, and by compression of the ipsilateral IJV, the ipsilateral PT is diminished, and there is no abnormality on the other side. SSS and SSWA usually occur on the right side because the right side is often the area of dominant drainage. Venous stenosis creates turbulent blood flow, resulting in a sound. The pathophysiological mechanisms behind this sound may be related to trans-stenotic gradient pressure (7, 8). The accelerated flow velocity caused by SSS may lead to SSWA because of higher pressures on the vessel wall and lower wall shear stress (9). SSWA amplifies the abnormal flow sounds due to SSS, further promoting PT. Some studies have suggested that it is SSWA that is the main determinant of PT occurrence (10). We believe that the PT in this case was caused by SSS. Because our careful review of all of the patient's imaging did not reveal anything about SSWA, we only identified SSS. Therefore, we hoped that by the first stent placement in SSS, by improving stenosis, decreasing blood flow velocity, and utilizing the shielding effect of the stent to decrease vascular tone transmission, we would temporarily leave the high JB untreated, as it has been shown that PT disappeared in some patients after improving stenosis while leaving the high JB untreated (11). In this case, the PT immediately disappeared after the first procedure, confirming the relationship between SSS and the PT.

However, the patient's PT reappeared after the third postoperative day when the stent was normal, suggesting that the PT was not only due to SSS but also possibly due to high JB and JBWD. Improvement of stenosis by stent placement alone was not sufficient to eliminate the PT symptoms in this patient. The top of the wall of the JB protruding into the internal auditory canal within 2 mm was considered a high JB. Similarly, a high JB generates an unstable vortex that produces a characteristic whistle sound and leads to oscillations in pressure fields (12). The absence of adventitia in the JB amplifies unusual sounds, which are then transmitted to the inner ear through the temporal bone, cochlea, and other structures. A high JB also obstructs venous blood return in the inner ear, causing edema of the auditory hair cells. This leads to abnormal discharge of the hair cells and irreversible damage to cochlear function, resulting in symptoms such as tinnitus and hearing loss. On the one hand, the eddy currents formed by the high JB can damage the vessel walls of the JB and on the other hand, the venous outflow laterality may generate a force that acts on the high JB, making it susceptible to mural fracture (13). In this way, the sound and shock produced by the blood vortex are transmitted through the JBWD into the inner ear or mastoid cavity. In this case, high-resolution CT showed that the JB had invaded the structures of the inner ear and caused JBWD. We embolized the high JB with spring coils so that no more blood flow passed through it, which prevented the production of an abnormal blood sound, and also prevented further structural damage and hearing damage due to the constant impingement of blood flow on the vessel wall of the JB through the blocking effect of the spring coils.

It is difficult to distinguish between SSWA and JBWD in terms of clinical symptoms, both may present with abnormal mastoid noises, hearing loss, vertigo, etc. However, the two can be differentiated by imaging tests such as high-resolution CT, where SSWA may be demonstrated as irregular sigmoid sinus bone wall or adjacent cranial cortical changes or thinning or disappearance of the sigmoid sinus superficial cortical bone wall or mastoid bubbles with direct sigmoid sinus contact, and JBWD may be demonstrated by high JB invasion into the inner ear structures and an incomplete thin bony jugular plate between the JB and the middle ear cavity (14). The special feature of this case is the coexistence of SSS and JBWD (Figure 1) and the different results of the two interventional procedures confirm that both etiologies contributed to the development of PT in the patient at the same time.

Reconstruction of the sigmoid sinus wall or the wall of the JB through some biomaterials such as bone cement or autologous soft tissues such as temporal fascia may also be a better approach, but one may face extensive thrombosis and IIH, and the autologous soft tissues may not be able to withstand the pressure of the blood flow (15), reconstruction needs to be comprehensively addressed at every point of the dehiscence, and any omission may result in the need for a second reconstructive surgery, and in addition, reconstructive surgery is an open procedure, which may cause more damage to the surrounding normal tissues. Embolization of the sigmoid sinus via spring coils or gelatin sponges is also a method of relieving PT symptoms, but it may lead to increased pressure in the dural sinus causing IIH as well as papilledema, which affects vision (16). Placement of a stent to improve SSS is a safer method that avoids the continuous washout of blood against the vessel wall, improves the direction of abnormal blood flow, and reduces the vibration of the vessel, while the stent acts as a shield to reduce the conduction of abnormal sounds, and also avoids the development of IHH because there is no loss of drainage from the normal sigmoid sinus (17–19).

However, intravenous stenting is a complex procedure with a significant complication rate, including in-stent stenosis, hematoma, in-stent thrombosis, and bracket detachment (20). To prevent thrombotic complications, guidelines state that anticoagulation needs to be resumed as soon as possible after neurointervention, with dual antiplatelet therapy(DAPT) being the preferred choice, and that the same patients need lifelong oral anticoagulants to avoid stent-related thromboembolism (21). We believe that DAPT anticoagulation should be used for 12 months after venous stenting (in the absence of coagulation abnormalities), Clopidogrel 75 mg plus Aspirin 100 mg is recommended. Also lifelong anticoagulants such as Dabigatran. This does increase the burden on the patient. In-stent restenosis(ISR) is also a problem that needs to be faced, as stent implantation causes damage to the vessel wall, which in turn causes an inflammatory response that can contribute to ISR, as evidenced by the fact that 3 out of 133 patients with stenting of the IJV and cerebral venous sinus developed intraluminal restenosis at an average follow-up of 33 months (22), therefore in-stent restenosis needs to be taken very seriously and needs further long-term follow-up is needed.

## 6 Conclusion

In conclusion, this case study suggests that PT may be related to changes in blood flow and pressure due to SSS and the high JB. A dehiscence in the wall of the JB or the wall of the sigmoid sinus may be crucial for the development of PT. Endovascular therapy may be the safer way to treat PT. The prevention of post-stenting complications and the effects of stenting on patients' hemodynamics require sustained attention. Noninvasive imaging of the intracranial vasculature can help identify possible causes of PT and localize the target vessel. By improving the hemodynamics of the vessel, the diagnosis and treatment of PT might be performed. However, this patient still experiences unresolved symptoms of other forms of tinnitus, which require further investigation to determine the underlying cause and find a resolution.

## Data availability statement

The original contributions presented in the study are included in the article/supplementary material, further inquiries can be directed to the corresponding author.

## Ethics statement

Written informed consent was obtained from the individual(s), and minor(s)' legal guardian/next of kin, for the publication of any potentially identifiable images or data included in this article.

## Author contributions

MX: Writing – original draft, Writing – review & editing. XD: Resources, Writing – review & editing. CZ: Resources, Writing – review & editing. TZ: Writing – review & editing. GW: Funding acquisition, Supervision, Writing – review & editing.
